# Causal Associations Between Obstructive Sleep Apnea and Hypertension: Evidence From a Bidirectional and Multivariable Mendelian Randomization Analysis

**DOI:** 10.1155/ijhy/8814506

**Published:** 2025-11-28

**Authors:** Shiyi Tang, Qiwei Li, Hua Yang, Yijun Liu

**Affiliations:** ^1^Department of Pediatric Otorhinolaryngology, West China Second University Hospital, Sichuan University, Chengdu, Sichuan, China; ^2^Department of Pediatric Pulmonology and Immunology, West China Second University Hospital, Sichuan University, Chengdu, Sichuan, China; ^3^Key Laboratory of Birth Defects and Related Diseases of Women and Children, West China Second University Hospital, Sichuan University, Ministry of Education, Chengdu, Sichuan, China

**Keywords:** association, hypertension, Mendelian randomization, obstructive sleep apnea

## Abstract

**Backgrounds:**

Obstructive sleep apnea (OSA) and hypertension both had significant impacts on human health. Whether OSA increases the risk of hypertension in relation to heredity remains unclear. We sought to clarify this issue using bidirectional Mendelian randomization (MR) analysis in large cohorts.

**Methods:**

A bidirectional two-sample MR was conducted to evaluate the potential causality between OSA and hypertension by selecting single-nucleotide polymorphisms (SNPs) as instrumental variables (IVs) from meta genome-wide association studies (mGWAS). The inverse-variance weighted (IVW) method was the main approach for data analysis to estimate the possible causal effects. Alternative methods such as MR-Egger, the MR pleiotropy residual sum and outlier (MR-PRESSO), and leave-one-out analysis methods were also performed as sensitivity analysis approaches. In order to adjust the confounding factors, a multivariable MR (MVMR) was also performed.

**Results:**

In the forward analysis, the IVW analysis demonstrated a significant association of OSA on increased hypertension risk (OR, 1.044; 95% CI, 1.012–1.076; and *p*=0.006), DBP value (β, 0.041; 95% CI, 0.012–0.071; and *p*=0.005), and hypertension of siblings risk (OR, 1.025; 95% CI, 1.003–1.049; and *p*=0.028), but after adjusting the confounding factors (BMI, smoking, and alcohol consumption), the association disappeared. The reverse analysis demonstrated the causal effect of hypertension of father on elevated OSA risk (OR, 3.803; 95% CI, 1.135–12.726; and *p*=0.030).

**Conclusions:**

Our forward analysis found the association between OSA and increased hypertension risk, DBP, and hypertension risk of siblings, but after adjusting the confounding factors, the association disappeared; on the reverse analysis, we found the causal effect of hypertension of father on elevated OSA risk.

## 1. Introduction

As one of the most common chronic diseases, hypertension is a leading cause of cardiovascular disease (CVD) and premature death worldwide [1]. It is estimated that the number of hypertensive people aged 30–79 has increased from 648 million in 1990 to 1.27 billion in 2019 [2]. Given the heavy burden of hypertension on society and individuals, early prevention and control of hypertension to reduce the risk of premature death from CVD is of great significance to improve the level of global public health [3]. Previous studies have shown that nongenetic risk factors for hypertension include physical inactivity, excessive salt in diet, overweight or obesity, smoking, and alcohol consumption [4], while accumulating evidence supported the promotive effect of obstructive sleep apnea (OSA) on hypertension [5–7].

Characterized by recurrent hypoxic episodes at night and subsequent sleep fragmentation due to complete or partial collapse of the upper airways, OSA has affected 936 million adults aged 30–69 years worldwide, including 425 million adults with moderate to severe OSA [8]. It is estimated that approximately 25% of hypertensive patients have OSA [9]. Nowadays, OSA has been regarded as an independent risk factor for hypertension [10]. However, the underlying mechanisms of blood pressure changes in OSA remain incompletely understood, and researchers are working to predict hypertension effectively in OSA patients [11]. On the other hand, although the relationship between OSA and hypertension has been recognized, the existing studies mainly focus on clinical data to illustrate this relationship, and whether this relationship is also meaningful in genetic level is unclear.

Mendelian randomization (MR) is an analytical approach that examines the causal effects of changeable exposure to diseases using human genetic variation [12]. Currently, common variants are widely used to investigate the potential causal relationship between risk factors and diseases using MR analysis. Based on data from genome-wide association studies (GWAS), a large number of new drug targets have been identified and validated, which can also assist in the clinical development phase of drugs [13].

Therefore, to determine the causal relationship between OSA and hypertension (which includes seven phenotypes: causal relationship between OSA and hypertension, DBP, SBP, hypertension of mother, hypertension of father, hypertension of siblings, and age of hypertension diagnosed), we performed a bidirectional MR analysis based on summary statistical results from GWAS data. Furthermore, in order to eliminate the potential confounding effect caused by common risk factors, we carried out a multivariable analysis to adjust the causal effect between OSA and hypertension. We believed that this study could fill in the gaps in the genetic relationship between OSA and hypertension.

## 2. Methods

**Figure 1:**
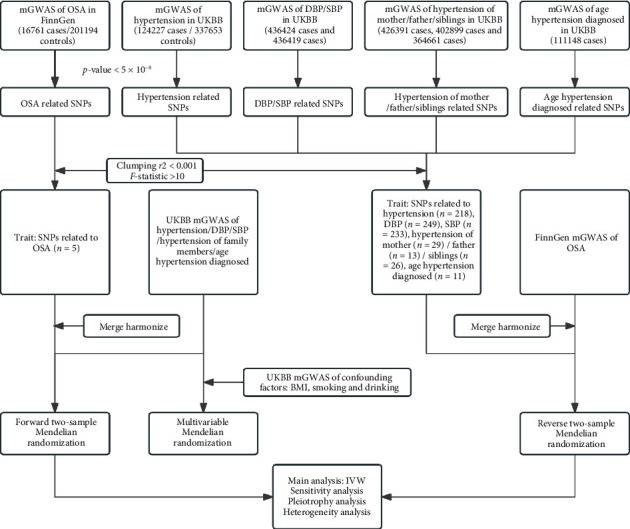
Flowchart of the Mendelian randomization analysis. mGWAS, meta-analysis of genome-wide association studies; OSA, obstructive sleep apnea; FinnGen, FinnGen consortium; UKBB, UK biobank; DBP, diastolic blood pressure; SBP, systolic blood pressure; SNPs, single nuclear polymorphisms; BMI, body mass index; IVW analysis, inverse-variance weighted analysis.

To analyze the causal relationship between OSA and hypertension, we conducted a bidirectional two-sample MR analysis first, for the phenotypes with statistical significance; we then performed a multivariable MR to adjust the effect of other possible risk factors. The design of our MR framework is illustrated in Figure 1.

### 2.1. Data Sources

#### 2.1.1. Ethical Approval

The genetic data in this study are obtained from the open access database GWAS, and no informed consent is needed for the application of data from GWAS. No details regarding individuals are reported in the study.

#### 2.1.2. GWAS of OSA

The genetic data for the risk of OSA were obtained from the most recent version of GWAS analyses of the OSA and controls in FinnGen individuals, which cohort contains 16761 OSA patients and 201194 controls [14]. The diagnosis of OSA was based on ICD-10 code G47.3 and ICD-9 code 3472A, which were obtained from the Finnish National Hospital Discharge Registry and the Causes of Death Registry [14]. The effects of all single-nucleotide polymorphisms (SNPs) were adjusted with age and sex.

#### 2.1.3. GWAS of Hypertension

The summary-level data of hypertension were obtained from the UK Biobank (UKBB) individuals, which provided the GWAS data of hypertension (124227 cases and 337653 controls), diastolic blood pressure (436424 individuals), systolic blood pressure (436419 individuals), hypertension of family members (including mother, father, and siblings with 426391, 402899, and 364661 individuals, respectively) and age hypertension diagnosed (111148 individuals) [15].

#### 2.1.4. GWAS of Other Risk Factors

Other risk factors included body mass index (BMI), tobacco smoking, and alcohol consumption, which had been proven to be associated with hypertension in genetic level [4, 16]. The BMI GWAS data were obtained from the UKBB individuals [15], which included 461460 individuals, and the tobacco smoking and alcohol consumption GWAS data also came from the UKBB with 33928 cases/302096 controls and 336919 cases/23807 controls, respectively. The details of all the GWAS data used in this research are shown in Table 1.

### 2.2. Statistical Analysis

#### 2.2.1. Instruments of Exposure

For the instrument variables (IVs), all chosen SNPs met the genome-wide significance criteria (*p* < 5 × 10^−8^). To ensure that the effect of SNPs on OSA and hypertension were related to the same allele, the effect direction was harmonized. Furthermore, we removed SNPs that were in linkage disequilibrium (r2 threshold < 0.001 within a 10 Mb window) from the outcome datasets and retrieved the remaining SNPs.

Then, we calculated the F statistic to assure there was no weak instrument bias (*F* > 10) and the *R*^2^ statistic to measure the explanation of the IVs on exposure. The *F* and *R*^2^ statistics were calculated using the following formula: *R*^2^ = 2 × EAF × (1 − EAF) × *β*2 and *F*=*R*^2^ × (*N* − 2)/(1 − *R*^2^) [17]. The statistical power was calculated using an online tool at https://cnsgenomics.com/shiny/mRnd/ [18].

#### 2.2.2. MR Analyses

To analyze the causal effects between OSA and hypertension, the inverse-variance weighting (IVW) method was adopted as the main analytical strategy [19]. To address variant heterogeneity and pleiotropic effects, five different two-sample MR approaches (MR-Egger, weighted median [WM], the MR pleiotropy residual sum and outlier (MR-PRESSO), simple mode, and weighted mode) were applied. When less than half of the weights came from invalid variants, the WM technique yielded effect estimates [20], and the MR-Egger technique produced consistent results even when up to 50% of the genetic variation was invalid [21]. The MR-PRESSO approach provides a corrective test by recognizing and deleting potentially pleiotropic outliers [22]. The statistically significant nonzero intercept of the MR-Egger intercept test indicated that the IVW results might be invalid because of horizontal pleiotropy [23]. Furthermore, the leave-one-out study was conducted to determine how eliminating one genetic variant from the MR analysis affected the results [24]. Concerning risk factors such as BMI, smoking, and alcohol consumption might disturb the causal effect between OSA and hypertension, a multivariable Mendelian randomization (MVMR) was performed to estimate the direct causal effect of OSA on the risk of hypertension. *p* < 0.05 was considered to indicate a statistically significant difference. R Version 4.2.2 (R Foundation for Statistical Computing, Vienna, Austria) with the two-sample MR (v0.5.6) and MR-PRESSO (v1.0) packages was used for all statistical analyses.

## 3. Results

### 3.1. Causal Effects of OSA on Hypertension Risk

**Figure 2:**
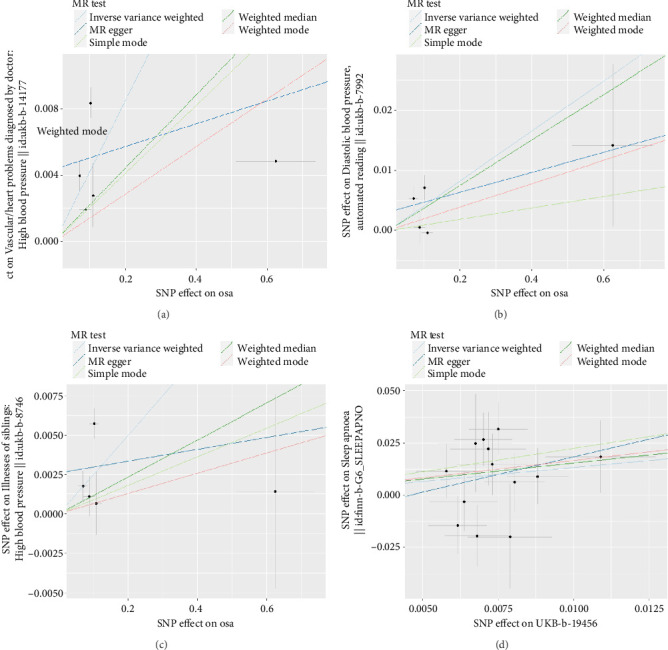
Scatter plot of the SNP effects on OSA and hypertension. (a) Scatter plot of OSA and hypertension. *X* axis: SNP effects on OSA, *Y* axis: SNP effects on hypertension. (b) Scatter plot of OSA and diastolic blood pressure. *X* axis: SNP effects on OSA, *Y* axis: SNP effects on diastolic blood pressure. (c) Scatter plot of OSA and hypertension of siblings. *X* axis: SNP effects on OSA, *Y* axis: SNP effects on hypertension of siblings. (d) Scatter plot of hypertension of father and OSA. *X* axis: SNP effects on hypertension of father, *Y* axis: SNP effects on OSA. The slope of each line corresponding to the estimated MR effect per method. MR, Mendelian randomization; SNP, single-nucleotide polymorphism; OSA, obstructive sleep apnea.

**Figure 3:**
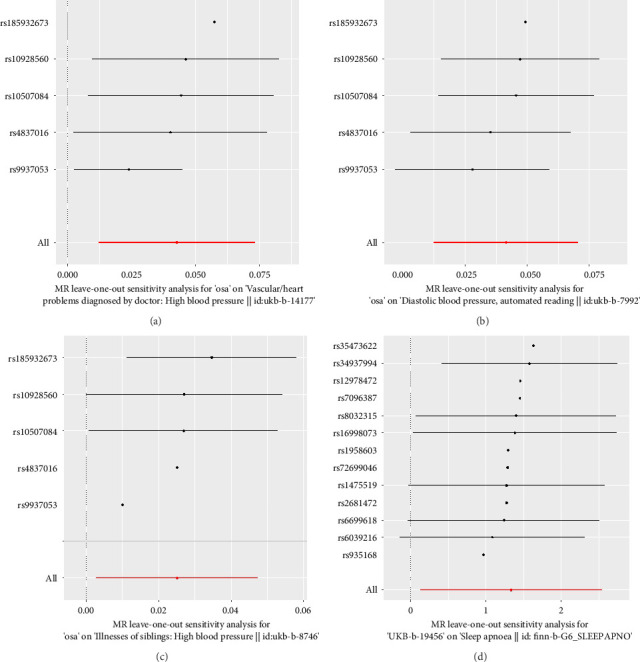
Leave-one-out plot. (a) MR sensitivity analysis for OSA and hypertension. (b) MR sensitivity analysis for OSA and diastolic blood pressure. (c) MR sensitivity analysis for OSA and hypertension of siblings. (d) MR sensitivity analysis for hypertension of father and OSA. MR, Mendelian randomization; OSA, obstructive sleep apnea.

In the IVW mode, we found that OSA had a significant association on hypertension (OR, 1.044; 95% CI, 1.012–1.076; and *p*=0.006). The WM mode also consistently revealed association between them (OR, 1.022; 95% CI, 1.004–1.041; and *p*=0.017). For diastolic blood pressure, we also discovered an association of OSA on it (IVW: β, 0.041; 95% CI, 0.012–0.071; and *p*=0.005; WM: β, 0.038; 95% CI, 0.007–0.069; and *p*=0.018). Also, for the OSA patients, their siblings are more likely to get hypertension (IVW: OR, 1.025; 95% CI, 1.003–1.049; and *p*=0.028). The scatter plots A, B, and C in Figure 2 showed the relationship between OSA and hypertension risk. The MR-Egger intercept test revealed no pleiotropic effects (*p*=0.249/0.432/0.373). We also applied leave-one-out analysis and failed to identify one SNP that substantially influenced the IVW estimate (Figures 3(a), 3(b), and 3(c)). The OSA did not appear to have a causal effect on the other 4 phenotypes of hypertension (SBP, hypertension of mother, hypertension of father, and age of hypertension diagnosed) (Table 2).

### 3.2. Causal Effects of Hypertension on OSA Risk

In the IVW mode, we found that hypertension of father had a causal risk effect on OSA (OR, 3.803; 95% CI, 1.135–12.726; and *p*=0.030). The scatter plot D in Figure 3 shows the relationship between hypertension of father and OSA risk. The MR-Egger intercept test revealed no pleiotropic effects (*p*=0.601), and the Cochran's *Q* test/MR-PRESSO global test revealed there were no significant heterogeneity for hypertension of father and OSA (all *p* > 0.10). We also applied leave-one-out analysis and failed to identify one SNP that substantially influenced the IVW estimate (Figure 3(d)). The other 6 phenotypes of hypertension did not appear to have a causal effect on OSA (Table 3). All of the F-statistic were above 10.

### 3.3. The Direct Causal Effect of OSA on the Risk of Hypertension

In the MVMR analysis, we adjusted the confounding factors such as BMI, smoking, and alcohol consumption in the causal effect of OSA on hypertension, but unfortunately, we did not find a causal relationship between OSA and hypertension after adjusting the effect of BMI, smoking, or alcohol consumption.

## 4. Discussion

Since OSA and CVD share several common risk factors, including obesity, gender, and age, the causal relationship between them has long been unclear [25]. The aim of this study was to explore the causal relationship between OSA and hypertension, and some intrinsic associations were successfully found.

It is theorized that the presence of OSA may severely cause a variety of physiological disturbances, including blood gas abnormalities, respiratory arousal, and surges of sympathetic nerve activity, which can produce the peak of blood pressure (BP) and heart rate [26]. Untreated OSA patients are exposed to nocturnal intermittent hypoxemia and hypercapnia, reoxygenation cycles, respiratory arousal and intrathoracic pressure fluctuations, and changes in the activity of the autonomic nervous system, so the risk of CVD increases through BP dysregulation, endothelial function, inflammation, oxidative stress, and metabolism [27]. In our forward analysis, we found there is a significant association between OSA and increased hypertension risk, which is consistent with the results of other recent researches [28, 29].

Furthermore, our forward research indicated a significant association between OSA and DBP, while the existence of OSA does not increase SBP. In our opinion, this result illustrates the influence from sympathetic nervous activity (SNA), which is closely linked to sleep. The effects of SNA on BP are deeply understood. To the best of our knowledge, SNA tend to elevate DBP preferentially, while elevations in SBP are mainly caused by atherosclerotic arteries [10]. Patients with OSA experience increased SNA, thus suffering elevated DBP. Another finding from our forward research is the association between OSA and increased hypertension risk of siblings, with no increased hypertension risk of parents. This result might be explained by many reasons including heredity, environment, and lifestyle. Genetic factors affecting CVD and OSA are likely to influence the susceptibility of an individual with OSA to develop CVD. Studies have shown that a variety of phenotypes are genetically related to the apnea hypopnea index, which is an indicator to assess the severity of OSA, including obesity, BP, and insomnia, which is consistent with those common genetic factors [30]. The fact implies that the siblings of an OSA patient might share the risk genetic factors of hypertension, but it needs to be determined with further application of large genetic databases.

In our study of reverse analysis, we found a causal effect of paternal hypertension on the elevated OSA risk, which means those whose father suffers from hypertension have a higher risk of OSA. On the one hand, large surveys about the OSA prevalence have reported a two-to three-fold higher prevalence in men than in women [31]. On the other hand, a genetic analysis including data from more than 217,000 individuals showed that the risk of OSA has a strong genetic component [14]. It is now evident that the development of hypertension is associated with a polygenic profile, where epigenetic markers are known to influence gene expression, leading to the development of the disease [32, 33]. Considering that hypertension and OSA share several risk factors, such as obesity, smoking, and alcohol consumption, the genetic predisposition of hypertension might lay influence on the incidence of OSA. However, some maternal factors have also been reported to be associated with a higher risk of hypertension [34]. Therefore, we suggested that the susceptibility of children with hypertensive fathers may have connection to the Y chromosome, which requires further analyses to be clarified.

Although our unadjusted analyses indicated a significant association between hospitalized OSA and hypertension, this association lost statistical significance upon comprehensive adjustment for key potential confounders, including BMI, smoking, and alcohol consumption. This result is consistent with another research [29]. The most plausible explanation for this phenomenon is the potent confounding effect of shared risk factors, particularly obesity (as reflected by BMI). Obesity is a well-established, major independent risk factor for both OSA [35] and hypertension [36]. Concurrently, obesity induces a cascade of pathophysiological changes: insulin resistance, dyslipidemia, sympathetic nervous system activation, renal dysfunction, and systemic inflammation; these are central to the development and progression of hypertension [37]. Therefore, the observed unadjusted association in our study and others is likely not indicative of a direct causal effect of OSA on hypertension but rather largely reflects the confounding influence of this powerful shared underlying factor, obesity. Smoking and alcohol consumption, while also adjusted for and known contributors to both conditions [38, 39], likely play a secondary but contributory role in this confounding structure. Consequently, our adjusted models, which rigorously accounted for these confounders, suggest that OSA may not be an independent causal driver of hypertension in this hospitalized cohort, with the association primarily mediated through shared anthropometric and lifestyle factors.

In contrast to previous analyses, our research included family history as a variable and managed to produce some interesting results. However, this analysis has some limitations. First, the databases we utilized were predominantly European, and the data we analyzed were Europeans. Consequently, our research findings are particularly suited to the European context. Second, even though age and sex are both risk factors for OSA, the gender difference diminishes as OSA patients get older [40]. Therefore, it would be useful for us to do further stratified analyses based on age and gender. Furthermore, our research was confined to genomic-level analyses, necessitating subsequent validation through more large cohort studies.

Here, we presented the causal association between OSA and seven hypertensive phenotypes. We believed our results could be beneficial to consider when designing other analyses and treatments for patients with OSA.

## 5. Conclusion

Our findings demonstrated that there is a significant association between OSA and increased hypertension risk, DBP, and hypertension risk of siblings, but after adjusting the confounding factors such as BMI, smoking, and alcohol consumption, the association appeared to disappear; on the reverse analysis, we found the causal effect of hypertension of father on elevated OSA risk.

## Figures and Tables

**Table 1:** Characteristics of GWAS data for each instrument variables.

Traits	Sample size (cases/controls)	Population	Consortium
OSA	16761/201194	European	FinnGen
Hypertension diagnosed by doctor	124227/337653	European	UKBB
DBP	436424	European	UKBB
SBP	436419	European	UKBB
Hypertension of mother	426391	European	UKBB
Hypertension of father	402899	European	UKBB
Hypertension of siblings	364661	European	UKBB
Age of hypertension diagnosed	111148	European	UKBB
BMI	461460	European	UKBB
Tabacco smoking	33928/302096	European	UKBB
Alcohol consumption	336919/23807	European	UKBB

*Note:* FinnGen, FinnGen consortium; UKBB, UK Biobank.

Abbreviations: BMI, body mass index; DBP, diastolic blood pressure; GWAS, genome-wide association studies; OSA, obstructive sleep apnea; SBP, systolic blood pressure.

**Table 2:** Forward causal relationships between OSA and hypertension.

Phenotype	nSNPs	OR/*β* (95% CI)^∗^	*p*	*Q* pval	Intercept pval	Global *p*
*Hypertension*						
IVW	5	1.044 (1.012, 1.076)	0.006	1.88E − 07		
MR-Egger	5	1.007 (0.952, 1.065)	0.826		0.249	
MR-PRESSO	5	1.037 (1.014, 1.061)	0.085			0.011
WM	5	1.022 (1.004, 1.041)	0.017			
Simple mode	5	1.021 (0.994, 1.049)	0.209			
Weighted mode	5	1.015 (0.999, 1.030)	0.143			

*DBP*						
IVW	5	0.041 (0.012, 0.071)	0.005	0.166		
MR-Egger	5	0.017 (−0.045, 0.078)	0.636		0.432	
MR-PRESSO	5	0.041 (0.012, 0.071)	0.049			0.257
WM	5	0.038 (0.007, 0.069)	0.018			
Simple mode	5	0.009 (−0.045, 0.064)	0.750			
Weighted mode	5	0.019 (−0.034, 0.072)	0.513			

*SBP*						
IVW	5	0.068 (0.015, 0.120)	0.012	< 0.001		
MR-Egger	5	−0.001 (−0.090, 0.089)	0.990		0.187	
MR-PRESSO	5	0.052 (0.003, 0.101)	0.129			0.018
WM	5	0.048 (0.006, 0.089)	0.024			
Simple mode	5	0.041 (−0.033, 0.115)	0.340			
Weighted mode	5	0.044 (−0.011, 0.098)	0.192			

*Hypertension of mother*						
IVW	5	1.017 (1.000, 1.034)	0.051	0.054		
MR-Egger	5	0.987 (0.964, 1.009)	0.333		0.059	
MR-PRESSO	5	1.017 (1.000, 1.034)	0.122			0.122
WM	5	1.022 (1.006, 1.039)	0.007			
Simple mode	5	1.022 (0.998, 1.047)	0.153			
Weighted mode	5	1.023 (0.998, 1.048)	0.142			

*Hypertension of father*						
IVW	5	1.011 (0.996, 1.025)	0.149	0.100		
MR-Egger	5	0.991 (0.968, 1.015)	0.523		0.166	
MR-PRESSO	5	1.011 (0.996, 1.025)	0.222			0.153
WM	5	1.005 (0.989, 1.021)	0.544			
Simple mode	5	0.999 (0.970, 1.029)	0.965			
Weighted mode	5	0.997 (0.965, 1.030)	0.855			

*Hypertension of siblings*						
IVW	5	1.025 (1.003, 1.049)	0.028	0.001		
MR-Egger	5	1.004 (0.959, 1.051)	0.881		0.373	
MR-PRESSO	5	1.016 (1.006, 1.027)	0.094			0.033
WM	5	1.012 (0.995, 1.029)	0.172			
Simple mode	5	1.009 (0.988, 1.030)	0.438			
Weighted mode	5	1.006 (0.988, 1.025)	0.535			

*Age hypertension diagnosed*						
IVW	5	−0.052 (−0.150, 0.045)	0.294	0.002		
MR-Egger	5	0.058 (−0.123, 0.239)	0.574		0.264	
MR-PRESSO	5	−0.042 (−0.076, −0.008)	0.136			0.029
WM	5	−0.030 (−0.112, 0.051)	0.470			
Simple mode	5	−0.041 (−0.171, 0.089)	0.573			
Weighted mode	5	−0.013 (−0.165, 0.139)	0.874			

*Note:* MR-PRESSO, MR pleiotropy residual sum and outlier.

Abbreviations: CI, confidence interval; DBP, diastolic blood pressure; IVW, inverse-variance weighted; nSNPs, number of single nucleotide polymorphisms; OR, odds ratio; OSA, obstructive sleep apnea; SBP, systolic blood pressure; WM, weighted median.

^∗^For the categorical variables in outcome, the causal effect was described with OR; for the continuous variables, the causal effect was described with *β*.

**Table 3:** Reverse causal relationships between OSA and hypertension.

Phenotype	nSNPs	OR (95% CI)	*p*	*Q* pval	Intercept pval	Global *p*
*Hypertension*						
IVW	218	1.897 (1.423, 2.528)	1.24E − 05	7.88E − 07		
MR-Egger	218	1.237 (0.558, 2.739)	0.601		0.260	
MR-PRESSO	218	1.674 (1.295, 2.165)	1.11E − 04			< 0.001
WM	218	1.490 (1.030, 2.154)	0.034			
Simple mode	218	0.699 (0.242, 2.017)	0.509			
Weighted mode	218	1.427 (0.621, 3.277)	0.403			

*DBP*						
IVW	249	1.102 (0.978, 1.241)	0.111	1.23E − 06		
MR-Egger	249	0.891 (0.630, 1.260)	0.515		0.202	
MR-PRESSO	249	1.141 (1.016, 1.281)	0.026			< 0.001
WM	249	1.069 (0.909, 1.256)	0.419			
Simple mode	249	1.696 (0.987, 2.912)	0.057			
Weighted mode	249	0.992 (0.676, 1.454)	0.965			

*SBP*						
IVW	233	1.084 (0.956, 1.228)	0.209	2.08E − 06		
MR-Egger	233	1.007 (0.695, 1.460)	0.969		0.683	
MR-PRESSO	233	1.083 (0.959, 1.222)	0.199			< 0.001
WM	233	1.077 (0.914, 1.270)	0.375			
Simple mode	233	0.990 (0.608, 1.612)	0.969			
Weighted mode	233	0.990 (0.625, 1.568)	0.967			

*Hypertension of mother*						
IVW	29	2.140 (0.878, 5.215)	0.094	0.020		
MR-Egger	29	2.137 (0.054, 84.536)	0.689		0.999	
MR-PRESSO	29	2.140 (0.878, 5.215)	0.105			0.023
WM	29	2.144 (0.763, 6.027)	0.148			
Simple mode	29	2.466 (0.373, 16.321)	0.357			
Weighted mode	29	2.207 (0.412, 11.833)	0.363			

*Hypertension of father*						
IVW	13	3.803 (1.135, 12.746)	0.030	0.258		
MR-Egger	13	29.407 (0.015, 56258.664)	0.399		0.601	
MR-PRESSO	13	3.803 (1.135, 12.746)	0.051			0.282
WM	13	4.644 (0.985, 21.908)	0.052			
Simple mode	13	9.504 (0.671, 134.693)	0.122			
Weighted mode	13	5.272 (0.565, 49.235)	0.170			

*Hypertension of siblings*						
IVW	26	2.868 (0.734, 11.213)	0.130	1.08E − 07		
MR-Egger	26	2.374 (0.008, 741.087)	0.770		0.948	
MR-PRESSO	26	1.619 (0.746, 3.514)	0.234			< 0.001
WM	26	1.472 (0.478, 4.531)	0.500			
Simple mode	26	1.008 (0.141, 7.209)	0.994			
Weighted mode	26	1.169 (0.234, 5.846)	0.850			

*Age hypertension diagnosed*						
IVW	11	0.814 (0.619, 1.070)	0.141	0.423		
MR-Egger	11	0.323 (0.111, 0.941)	0.068		0.114	
MR-PRESSO	11	0.814 (0.619, 1.070)	0.171			0.403
WM	11	0.812 (0.560, 1.177)	0.272			
Simple mode	11	0.915 (0.511, 1.638)	0.771			
Weighted mode	11	0.842 (0.507, 1.398)	0.521			

*Note:* MR-PRESSO, MR pleiotropy residual sum and outlier.

Abbreviations: CI, confidence interval; DBP, diastolic blood pressure; IVW, inverse-variance weighted; nSNPs, number of single nucleotide polymorphisms; OR, odds ratio; OSA, obstructive sleep apnea; SBP, systolic blood pressure; WM, weighted median.

## Data Availability

The GWAS datasets generated and/or analyzed during the current study are publicly available.
